# Synthesis of highly fluorescent carbon quantum dots from rubber seed shells for the adsorption and photocatalytic degradation of dyes

**DOI:** 10.1038/s41598-023-40069-w

**Published:** 2023-08-07

**Authors:** Nurul Umairah M. Nizam, Marlia M. Hanafiah, Ebrahim Mahmoudi, Abdul Wahab Mohammad

**Affiliations:** 1 Department of Earth Sciences and Environment, Faculty of Science and Technology, Universiti Kebangsaan Malaysia, 43600 Bangi, Selangor Malaysia; 2 Centre for Tropical Climate Change System, Institute of Climate Change, Universiti Kebangsaan Malaysia, 43600 Bangi, Selangor Malaysia; 3 Department of Chemical and Process Engineering, Universiti Kebangsaan Malaysia, 43600 Bangi, Selangor Malaysia; 4 Chemical and Water Desalination Engineering Program, College of Engineering, University of Sharjah, Sharjah, United Arab Emirates

**Keywords:** Chemical engineering, Environmental sciences, Nanoscale materials

## Abstract

The potentials of biomass-based carbon quantum dot (CQD) as an adsorbent for batch adsorption of dyes and its photocatalytic degradation capacity for dyes which are congo red (CR) and methylene blue (MB) have been conducted in this study. The CQDs properties, performance, behaviour, and photoluminescence characteristics were assessed using batch adsorption experiments which were carried out under operating conditions including, temperature, pH and dosage. The morphological analysis revealed that CQDs are highly porous, uniform, closely aligned and multi-layered. The presence of hydroxyl, carboxyl and carbonyl functional groups indicated the significance of the oxygenated functional groups. Spectral analysis of photoluminescence for CQDs confirmed their photoluminescent quality by exhibiting high excitation intensity and possessing greenish-blue fluorescence under UV radiation. The removal percentage of the dyes adsorbed for both CR and MB dyes was 77% and 75%. Langmuir isotherm and pseudo-second-order models closely fitted the adsorption results. Thermodynamics analysis indicated that the adsorption process was exothermic and spontaneous, with excellent reusability and stability. The degradation efficiency of CQDs on both dyes was more than 90% under sunlight irradiation and obeyed the first-order kinetic model. These results demonstrated CQDs to be an excellent adsorbent and outstanding photocatalyst for organic dye degradation.

## Introduction

Anthropogenic activities that cause severe environmental pollution are not restricted to the textile industry. Other production sectors, such as pigment, paper and pulp, and food industries, have also been impacted^1–3^. Various chemicals are utilized during the processing stages, generating effluents and other associated pollutants such as heavy metal ions. The environmental pollution attributed to the discharge of dyes and metal ions poses a detrimental effect on the flora and the fauna^4,5^. Moreover, different studies have extensively reported that human beings and other life forms are susceptible to various health risks with dangerous consequences to the exposed population due to contact with dye molecules and heavy metal ions, even at low concentrations^6–8^. For instance, methylene blue (MB), also known as a cationic dye, has been broadly used for the dying of cotton, hemp, silk, and paper due to its inherent water solubility and excellent colour stability. For this reason, MB tends to exist and become persistent in water for a relatively long time without being degraded. Through daily contact, especially by washing MB-dyed materials, diseases such as cyanosis, vomiting, eye irritation and dermatitis have been widely reported^9–11^. In the same context, congo red (CR), an anionic dye for the dyeing of fabrics, silks, hemp, and papers. The release of residual concentration of these dyes in water sources has accelerated environmental risks and danger to human health^12–14^. Therefore, decontamination techniques to minimize poisonous pollutants' impact, including cationic and anionic dyes, is a critical research topic. The adverse effects of dye pollution concentration from wastewater streams can be reduced using numerous technologies, including membrane filtration^15–17^, electrochemical reduction and ion exchange^18–20^, coagulation and flocculation^21,22^, photocatalytic degradation^23–28^ and adsorption^2,12,13,29–31^.

Comparatively, due to its ease of use at a low cost, low energy consumption, high treatment efficiency, and affordable precursory materials as a source of adsorbents, the adsorption process is one of the most promising and cost-effective techniques^11,32,33^. In recent years, the synthesis of new generation carbon quantum dots (CQDs) from waste precursors has gained more remarkable attention than other quantum dots^11,33^, especially in wastewater treatment, to enhance the removal of pollutant species from solution due to its facile preparation method^5,34–37^. The advanced properties of CQDs have become a hotspot of research interest due to their intrinsic specific surface area, low toxicity, tuneable luminescence, significant solubility in aqueous solution, and high photostability^4,38–41^. Due to its remarkable potential, the application of CQDs has been extended to other areas, including chemical sensing, biosensing, bioimaging, photocatalysis and nanomedicine^5,35–37^. CQDs have been applied to exploit their intriguing potentials, such as excellent light absorption ability, abundantly available surface, high dispersibility, excellent photostability and tunable bandgap structure. It has been used as an effective alternative for the photodegradation of pollutants compared to the conventional quantum dots, which is very expensive and toxic.^42,43^. Several methods of synthesizing CQDs have been proposed in the past few years and have shown to be effective with excellent photocatalytic properties^44^. Some of these methods include arc laser ablation^45^, hydrothermal^32,46,47^ and pyrolysis techniques^48^ as shown in Table 1 including their applications. However, some of these methods suffer challenges and difficulties in the synthesis approach, which in most cases are rather tedious. Furthermore, complex instrumental setup, complexity in chemical reactions, limited spectral efficiency and low product yield have limited their wide application.

**Table 1:** Fabrication methods of CQDs reported in the literature.

Method	Details	Applications	References
Hydrothermal method	CQDs are synthesised in a high-temperature and high-pressure aqueous environment	Bioimaging, biosensing, drug delivery	^32,46,47^
Microwave-assisted method	CQDs are synthesised using microwave radiation	Bioimaging, phototherapy, photocatalysis	^50–53^
Solvothermal method	CQDs are synthesised in a high-temperature and high-pressure organic solvent	Biomedical imaging and sensing, energy storage	^54–56^
Electrochemical method	CQDs are synthesised via electrochemical reaction	Electrochemical sensing, bioimaging, photocatalysis	^57,58^
Pyrolysis method	CQDs are synthesised by thermal decomposition of organic precursors	Sensing, bioimaging, photocatalysis	^48,59^
Laser ablation method	CQDs are synthesised by irradiating a carbon target with a laser beam in an aqueous environment	Bioimaging, biosensing, drug delivery	^45,60^
Photochemical method	CQDs are synthesised using light radiation	Bioimaging, photocatalysis	^61,62^

Green synthesis using rice husk wastes for the hydrothermal synthesis of CQDs has been reported by Hui et al.^49^. The result indicated that the enhancement of the photoluminescence properties and tunable band gap influenced the effective degradation of MB and Cu (II) ions which was achieved by doping CQDs with nitrogen and bismuth. Nugraha et al.^41^ synthesized composite of tungsten oxide/amino-carbon quantum dots derived from sugarcane bagasse using a hydrothermal method. Although the fabricated CQDs enhanced the degradation of MB by 84.61%, however, the combination of tungsten oxide with the CQDs produced from sugarcane bagasse enhanced MB's degradation efficiency by 92.93%. Recently a study has synthesized nitrogen-CQDs using lignin extracted from empty fruit bunch (EFB) and was used as a photocatalyst for the degradation of MB and malachite green (MG) by Rani et al.^23^. It was revealed that nitrogen doping to the CQDs surface improved the photostability, photo-chemical active site and quantum yield of CQDs. Multifunctional groups improved the performance of photocatalytic degradation in the presence of other functional groups. Similarly, Sabet et al.^46^ have investigated metal ions adsorption performance of nitrogen-doped carbon quantum dots derived from grass. The results showed astonishing adsorption capabilities where 75% of Pb^2+^ and 37% of Cd^2+^ were removed from the aqueous solution. The outstanding adsorption performance of the CQD has opened a new book in the field of multifunctional adsorbents. Previous studies have demonstrated that better photocatalytic degradation efficiency, which can result in improved degradation of dyes and metal ions, can be achieved by doping or combining CQDs with metal oxides. To date, only few studies have been conducted on the fabrication of CQDs from graphitized sources via a facile synthesis route for the photocatalytic degradation and adsorption of dyes from the wastewater stream.

Thus, the present study intends to fabricate highly fluorescent zero-dimension CQDs from rubber seed shells (biomass waste) with the aim of its utilization as an effective photocatalyst and adsorbent for the degradation and adsorption of dyes from solution. Based on their ability to adsorb CR and MB dyes from an aqueous solution, the fabricated CQDs' adsorption effectiveness and photocatalytic degradation capability were assessed. Through the analysis of X-ray diffraction (XRD), Raman spectroscopy, Fourier-transform infrared spectroscopy (FTIR), nano particle size analyzer, field emission scanning electron microscopy (FESEM), photoluminescence spectrophotometer, transmission electron microscope (TEM), and zeta potential, the characterization of the synthesized CQDs and their potential adsorptive capacity were studied. Finally, evaluations were made of modelling studies utilizing equilibrium isotherm, kinetic, and thermodynamic research.

## Result and discussion

### Characterization of CQDs

#### FTIR spectroscopy, Raman spectroscopy, Zeta Potential and XRD

**Figure 1:**
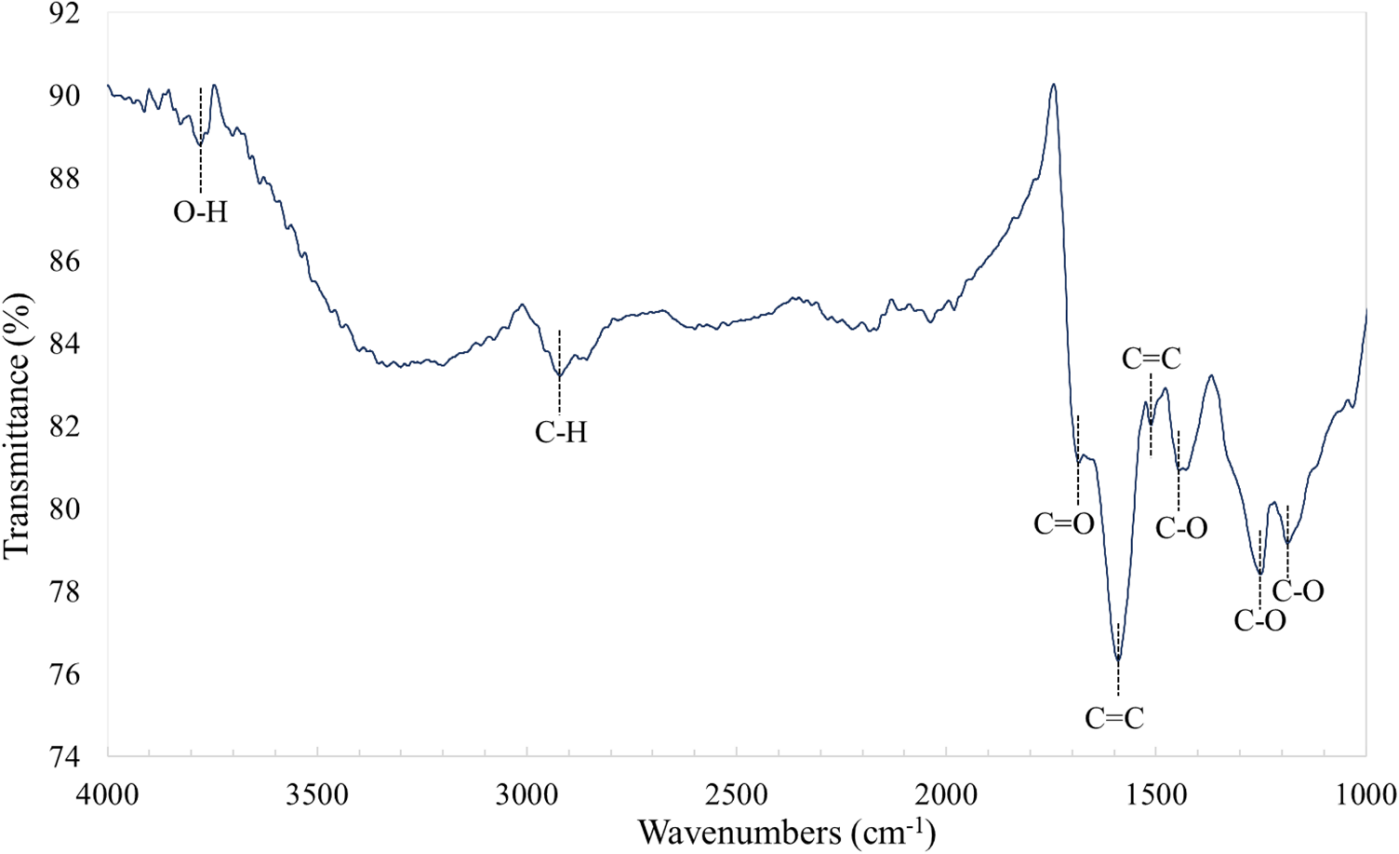
FTIR spectra of CQDs adsorbent.

**Figure 2:**
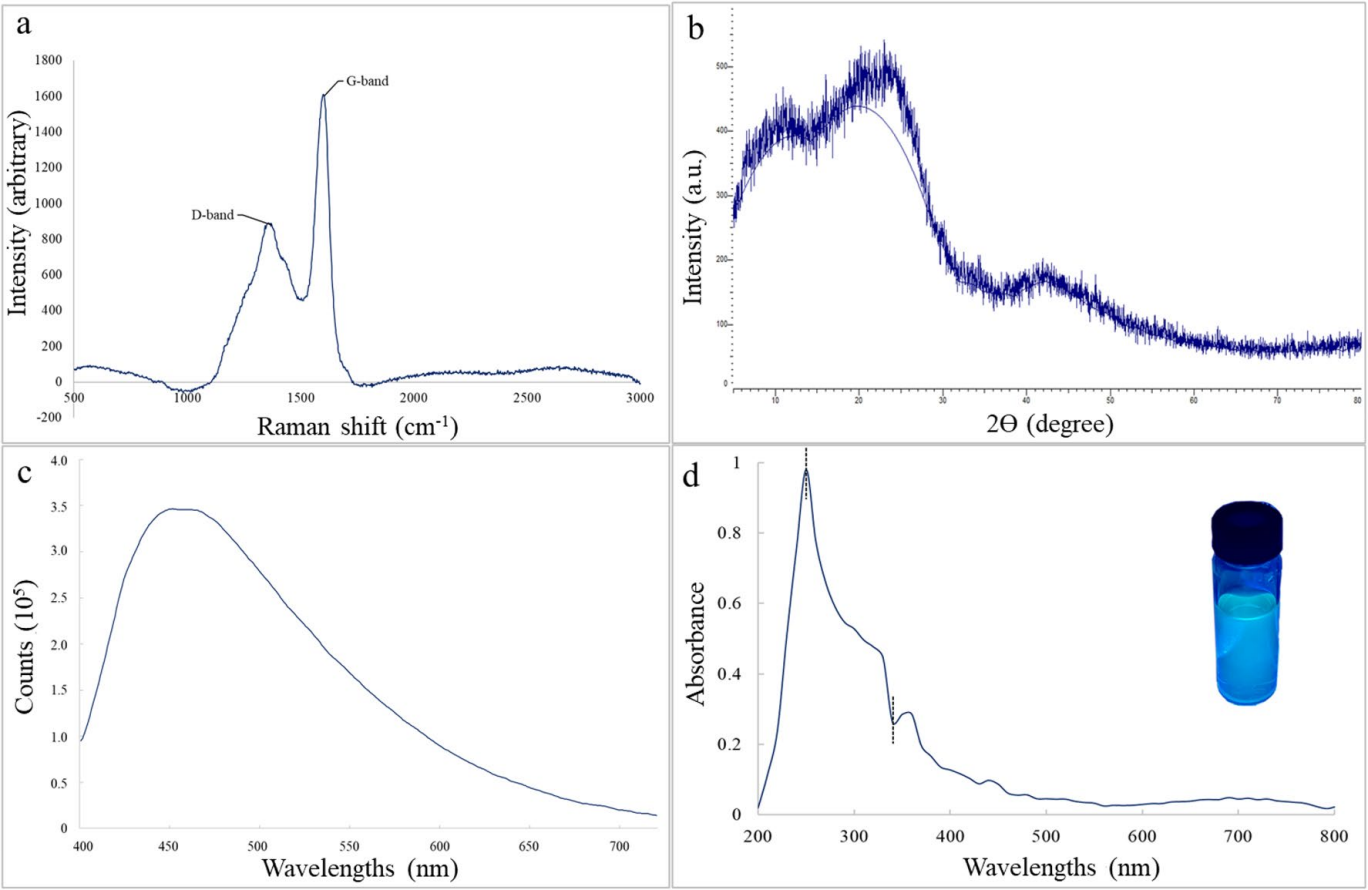
(**a**) Raman spectroscopy; (**b**) XRD spectra; (**c**) photoluminescence spectra of CQDs; and (**d**) UV spectra of CQDs with the image of its fluorescent colour (exposed under a 395 nm UV lamp).

The analysis of FTIR structural bands, Raman spectroscopy and XRD pattern of the CQDs adsorbents are shown in Figs. 1 and 2, respectively. The recognition of functional groups in the fabricated CQDs was obtained from the FTIR spectra (Fig. 1). The findings showed that the main and active functional groups that were on the CQDs structures were carboxyl, hydroxyl, and carbonyl groups which bounded to the aromatic ring^23^. The wave numbers around 1590.25 and 1513.3 cm^−1^ were attributed to the presence of C=C groups. A peak centred around 3743.45 cm^−1^ resembles to the O–H stretching vibrations while the adsorption spectra equivalent to 1444.89, 1250.61 and 1186.5 cm^−1^ can be relate to the C–O groups stretching. These peaks, which revealed an abundance of carboxyl, hydroxyl and carbonyl groups, indicated that oxygenated functional groups significantly influence the CQDs' functional properties^13,15,23^.

Figure 2a presents the Raman spectroscopy analysis of the carbon quantum dots (CQDs). The D band peak indicates that the CQDs possess inherent properties as a superior adsorbent. However, the material still exhibits defects as observed in the spectrum. The equivalent value of the I_D_/I_G_ ratio close to one proved that CQDs exhibited multilayer structures. However, the ascribed G band indicated that the crystalline structures of CQDs originated from phonon models. The presence of both peaks of CQDs confirmed the availability of the lattice distortions^12,13^. The XRD spectrum (Fig. 2b) at 25° signified a broad diffraction peak indicating structural crystal planes, which describes the presence of an amorphous peak of CQDs. The spectra of CQDs were found at 42°, suggesting that the interlayer spacings were raised due to the availability of oxygen functionalities^10,13^. Zeta potential values of 45.3 mV were attained. The presence of hydroxyl groups on the CQDs surface may have contributed to the negative charges, which represented the net charge of the scattering object of the CQDs^12,13^. The zeta values for both adsorbents indicated a stable colloidal dispersion in which all of the particles tend to resist one another, preventing the particles from aggregating, coagulating, or both^15,23^. Higher zeta values showed more porous surfaces and more stable colloidal dispersion^13^.

#### Photoluminescence spectroscopy and UV–vis spectrophotometry

The fluorescent qualities of the CQDs were observed by photoluminescence spectroscopy spectrophotometer (PL) to study the charge, its migration, and optical characteristics. CQDs photoluminescence is highly influenced by the excitation wavelengths, at which the intensities initially increased and subsequently, it was observed to decline gradually. From Fig. 2c, it was revealed that the excitation wavelength of CQD adsorbent was achieved at 448 nm, which implies that the luminescent properties were primarily attributed to the bandgap transitions^38–40^, which were observed to correspond to the surface defects existing in the CQDs, supporting the findings of Raman spectroscopy results. The sp^2^ hybridization of carbon clusters, along with the slight differences in particle size distribution, may also be attributed to the influence of the fluorescence aspects of the CQDs^12,35,37,63,64^. Moreover, the high excitation intensity of the CQDs subsequently resulted in higher energy emissions by the material, which influenced its luminescent properties.

The optical characteristics of the adsorbent were investigated using UV–vis adsorption analysis, shown in Fig. 2d. The results revealed that CQDs exhibited the peaks of characteristic transition of 200–450 nm wavelength. Meanwhile, it was indicated that the sunlight was absorbed in the visible and ultraviolet spectrums. The C=C bonds transition from the sp^2^ carbon bonds and aromatic π could be associated with the optical absorption peak of CQDs at 250 nm. The transitions of CO–H and C=O groups during the oxidation of CQDs corresponding to an equivalent peak at around 340 nm indicated the occurrence of oxygen-functionalized groups^12^. An observable greenish-blue fluorescence of the CQDs, as indicated in Fig. 2d, signified the exposure of fabricated CQDs to UV-light irradiation. The luminescence features of the quantum dots tend to create an enhanced photocatalytic redox reaction by promoting electrons and hole migration rates which can react with oxygen molecules to generate radical molecules^12,36^.

#### SEM, EDX mapping and TEM

**Figure 3:**
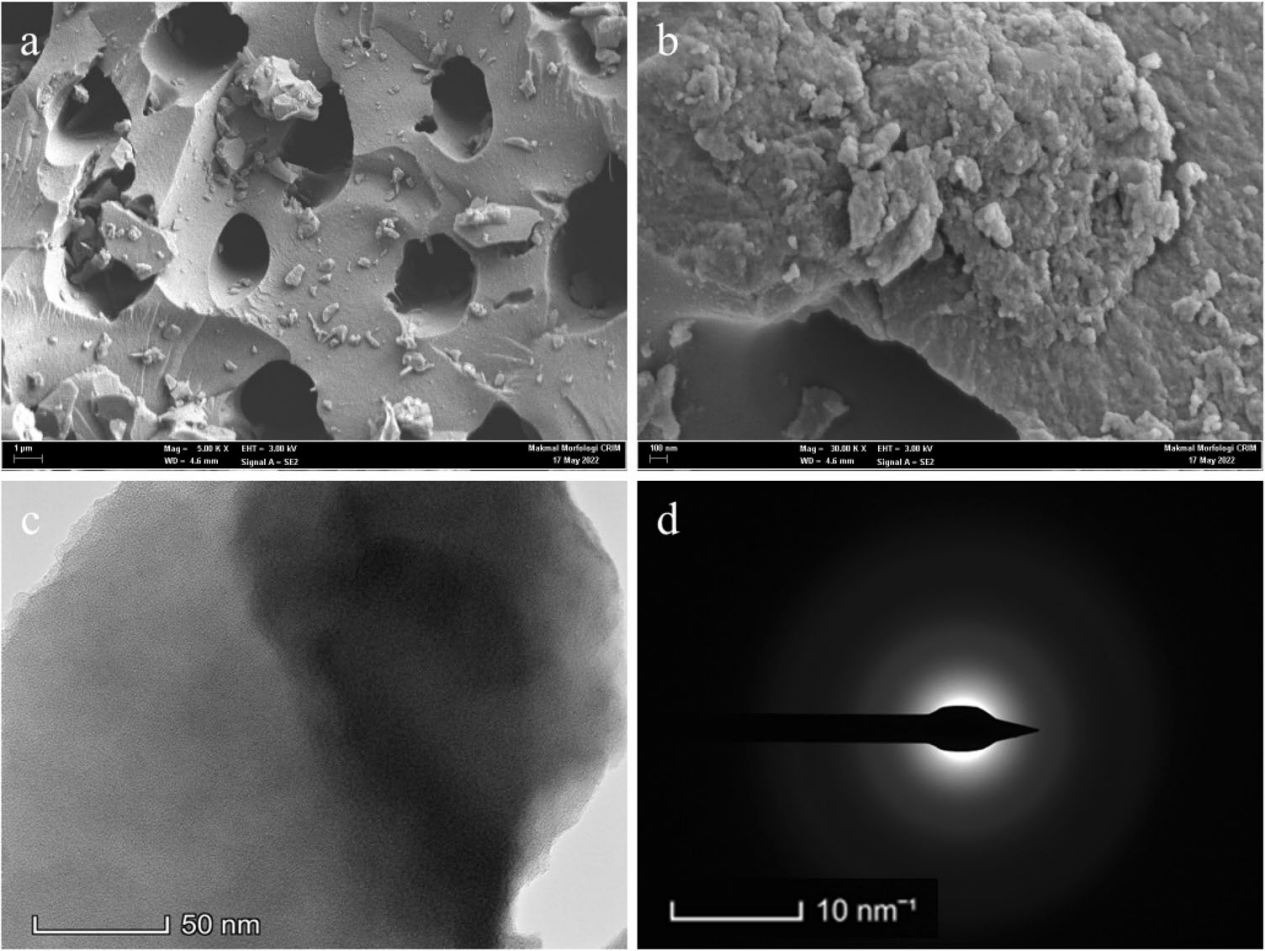
SEM images of; (**a**) morphology surface of CQDs at ×500 magnification; (**b**) morphology surface of CQDs at ×50 magnification; (**c**) TEM images for CQDs adsorbent in 50 nm scale, and (**d**) SAED pattern.

**Figure 4:**
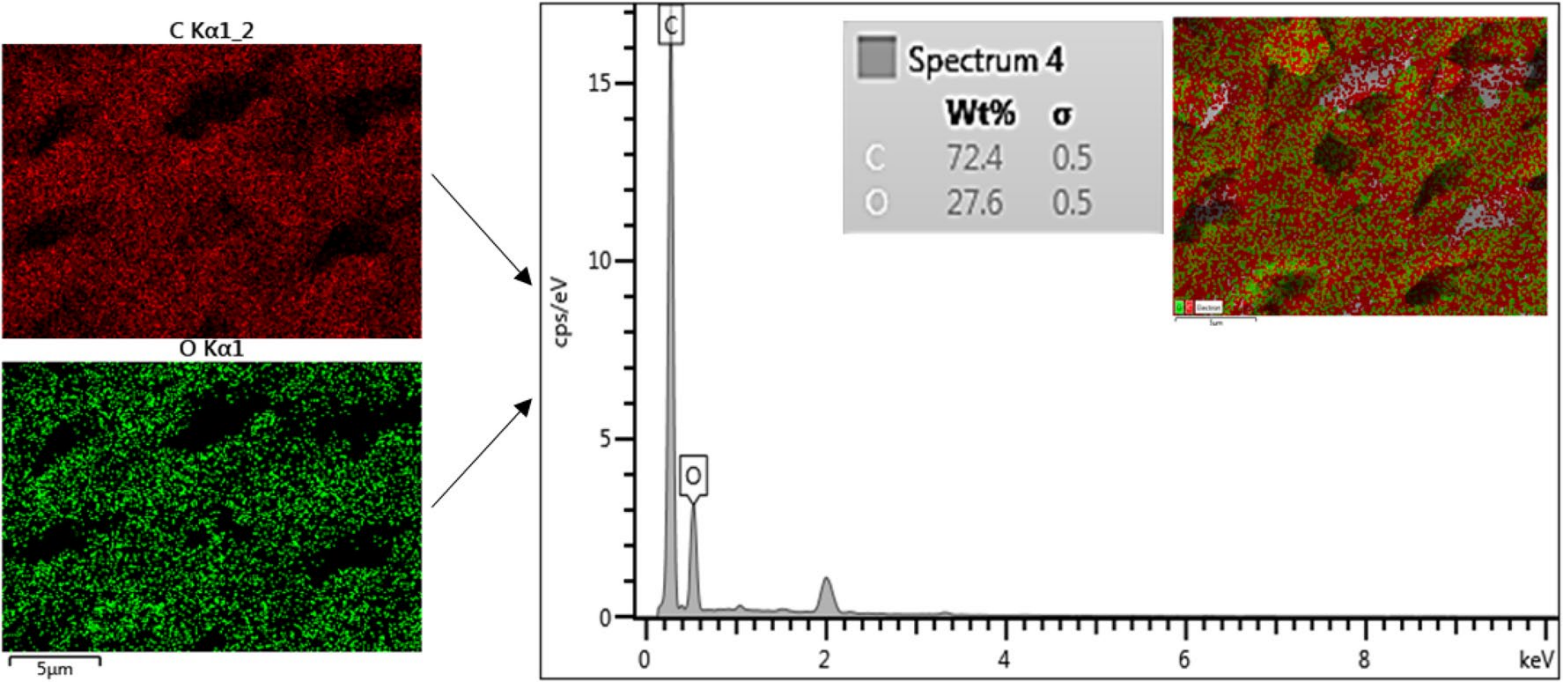
EDX mapping and analysis of elements on the surface of CQDs.

The distribution of particle size and surface morphologies of CQDs obtained at 500× magnification are indicated in Fig. 3a. The micrograph revealed relatively rough and uneven pore structures of CQDs across the boundaries, indicating a heterogeneous interfacial surface for dyes' adsorption and photocatalytic degradation. In some regions, the pore distribution was slightly uniform with closely aligned porous surfaces, thereby creating more tendency for the adsorption of the contaminants on the surface of the CQDs^30^. The aggregation of particles on some CQDs' surfaces, as observed in Fig. 3b, may likely be due to the tightly packed nano-sized particles of CQDs across the boundaries indicating that more available porous sites were created for the attachment of pollutants on the surface of CQDs^12^. Moreover, the EDX mapping results as shown in Fig. 4 indicated that vast homogeneous dispersion of carbon and oxygen elements were the predominant features on the surface of CQDs, revealing a more signified composition of wt% carbon compared to oxygen elements. A considerably high composition of oxygen elements in the adsorbent leads to enhanced adsorption efficiency as a result of the interfacial region's strong inclination to contain more broken π–π bonds. The CQDs were highly oxidized as a result of the high composition of oxygen atoms, thereby creating more active sites on their surface^12,13,30^. TEM images revealed that the fabricated CQDs’ surface morphology, as shown in Fig. 3c, exhibited less transparent layers confirming that the CQDs were a multilayer adsorbent rather than a single-layered carbon dot. The crystalline form of CQDs was further shown by the bright spots and diffuse rings in the selected area electron diffraction (SAED) pattern (Fig. 3d). Based on this, it can be inferred that the CQDs could be linked to the sp^2^ graphitic carbon and the diamond-like sp^3^ carbon diffraction plane.

### Adsorption of organic dyes

Analysis was carried out to determine the synthesized CQDs' efficiency for removing CR and MB under the adsorption and photocatalytic processes. For the purpose of assessing the adsorptive ability of the synthesized CQD adsorbents, equilibrium batch adsorption studies were carried out.

#### Adsorption of organic dyes on CQDs

**Figure 5:**
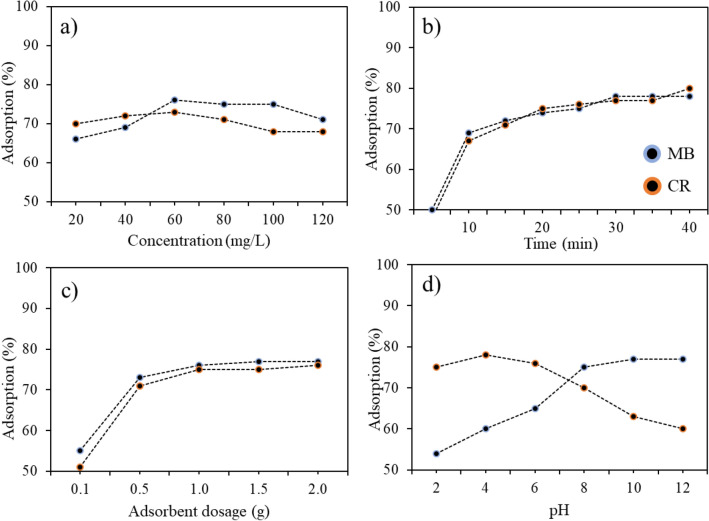
Optimization parameters of the adsorption for CR and MB, (**a**) initial concentration; (**b**) time; (**c**) adsorbent dosage; and (**d**) pH on the adsorption by CQD.

Several process parameters, such as initial concentration at the interval of 20–120 mg L^−1^ within contact time of 5–40 min using carbon dots dosage of 0.1–2.0 g at pH of 2–12 and temperature of 298–323 K, were examined to establish the optimum conditions to enhance the removal efficiency of CQDs for the adsorption of CR and MB. It was revealed that under the optimum condition of other operational parameters, the percentage removal of CR and MB increased when the initial concentration amplified from 20 to 60 mg L^−1^ (Fig. 5a), which indicated that the dye diffusion molecules into the pores and voids of the CQDs was not inhibited regardless of the increase in the dye solutions’ initial concentration. In the initial adsorption stage, for first 10 min, the adsorption rate of CQDs steadily increased for the removal of MB and CR until 40 min of contact time was reached (Fig. 5b). At the beginning of adsorption, more vacant functional groups and active sites were found on the surface of CQDs, influencing the absorption capacity^12,13^. These active sites generated extra adsorption sites for the CR and MB molecules in the solutions to adsorb to the CQDs at the initial phase of adsorption. After achieving equilibrium conditions, the surface of the CQDs was found to be saturated, thereby inhibiting further adsorption of CR and MB onto the adsorbent, maintaining the uptake capabilities. The effectiveness of CQDs, indicated in Fig. 5c, was also assessed by maximizing the adsorbent dose under given circumstances for other operating parameters. With a dosage of 500 mg, the uptake capacity of CQDs increased significantly for the adsorption of dyes. As adsorbent dosages were increased, constant uptake capabilities were achieved, indicating that there were sufficient active sites in the adsorbents at low doses to adsorb MB and CR^13^.

The impact of pH on the optimum adsorptive capability further highlighted the importance of the hydrogen bonds, hydrophobic bonds and π–π interactions on the surface of the CQDs (Fig. 5d). Additionally, the solution’s pH impacted the adsorption efficiency in relation to the surface charge and ionization of the adsorbent which enhanced the interface amongst the adsorbent and adsorbate^12,13,15,23,46^. In the acidic solution at pH levels between 4 and 6, high amount of H^+^ ions in the aqueous medium, which decreased the adsorption efficiency of cationic dye (MB), was achieved^65^. Moreover, the amount of H^+^ ions in the medium decreased as the pH of the aqueous solution increased, which improved the efficiency of the adsorption process. The anionic dye (CR), alternatively, had excellent adsorption effectiveness at low pH (4), in which may have been due to the limited competitive interactions amongst the H^+^ ion, the adsorbent surface and the adsorbate^66^. At a pH of 4, molecular and anionic forms coexist in roughly equal amounts, while pH values higher than 4 favoured an anionic species' predominance, resulting in CR dye producing polar groups at pH values of 4 and beyond. Therefore, these negative-charged groups influenced the adsorption of the dye through electrostatic interaction with CQDs, which acted as a catalyst and become negatively charged in an alkaline environment and positively charged in an acidic environment^13^. To sum up, the oxygen-containing functional groups on CQDs generally significantly impact the adsorbent surface. This was reflected in the adsorptive capacity of CQDs to remove CR and MB.

### Sorption isotherms

The numbers from the adsorption isotherms were fitted by the Freundlich, Langmuir and Dubinin–Radushkevich (D–R) isotherm models, as summarized in Table 2. The adsorption of CR and MB onto the surface of CQDs was best described by the Langmuir model, with R_2_ = 0.999, has a better correlation coefficient than Freundlich and D–R isotherm models. It was established that the monolayer adsorption mechanism, as defined by the Langmuir model, preferred better adsorption efficiency of CQDs for the removal of CR and MB. The CQDs efficiency was demonstrated by the Q_max_ values (maximum adsorption capability), which were substantially impacted by oxygen-derived functional groups on the adsorbent–adsorbate interaction's active sites. Due to the existence of CQDs' active functional groups, the π–π interaction and electrostatic interaction between the adsorbents and adsorbates are the best ways to explain the uptake capacity^67^.

**Table 2:** Isotherm models values for the adsorption of CR and MB by the CQDs.

Models	Parameter	MB	CR
Freundlich	k (mg g^−1^)	0.37	0.45
	1/n	0.68	0.62
	R^2^	0.94	0.92
Langmuir	Q_max_ (mg g^−1^)	451.54	375.94
	R^2^	0.99	0.98
Dubinin-Radushkevich	Q_max_ (mg g^−1^)	99.56	70.65
	E (kJ mol^−1^)	1.01	1.45
	R^2^	0.75	0.84

### Adsorption kinetics

The experimental values were fitted by the PSO, PFO, intraparticle diffusion (ID) and Elovich models to evaluate the kinetic mechanism of the adsorption process. The reason is to comprehend more information on the adsorption process of the dyes onto the fabricated CQDs. The kinetic evaluation results are shown in Table 3. The linear fit (R^2^) correlation coefficients indicated that the dyes adsorbed by the CQDs suited better with the PSO equations. These results showed that the adsorption process was chemically controlled (chemisorption)^12,15^, with better adsorption and increased bonding, the adsorbent and adsorbates interact more effectively. Furthermore, the adsorption capacity (q_e_) demonstrated an absolute agreement with the experimental results, suggesting that the PSO model, as opposed to the PFO model, better captured the nature of CR and MB adsorption onto the CQDs.

**Table 3:** Kinetic models values for the adsorption of CR and MB on CQDs.

Kinetic model	Parameter	MB	CR
Pseudo-first-order	R^2^	0.73	0.66
	q_e_ (mg g^−1^)	10.66	9.54
	k_1_ (min^−1^)	0.1	0.09
Pseudo-second order	R^2^	0.99	0.98
	q_e_ (mg g^−1^)	29.29	25.47
	k_2_ (g mg^−1^ min^−1^)	0.16	0.09
Intraparticle diffusion	K_ID_	1.87	1.56
	C	1.48	2.14
	R^2^	0.98	0.97
Elovich	α (mg g^−1^ min^−1^)	0.78	1.78
	β (g mg^−1^)	0.91	0.93
	R^2^	0.95	0.93

### Adsorption thermodynamics

**Figure 6:**
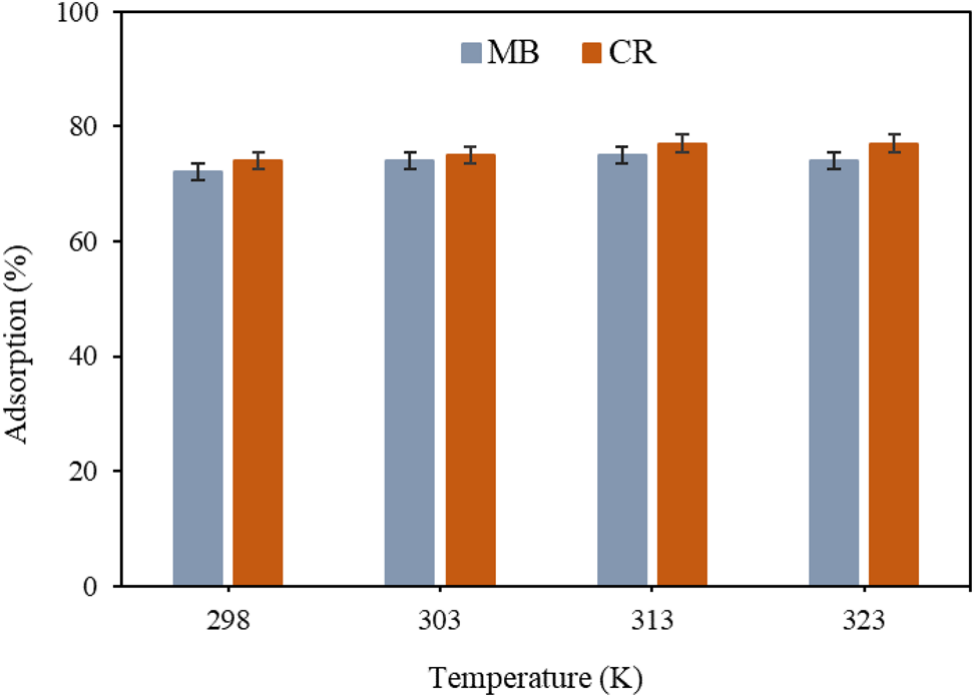
Optimization parameter for the adsorption of CR and MB through temperature of the adsorption by CQDs.

The parameters of thermodynamics (ΔG, ΔH and ΔS) and effects of temperature were evaluated according to Eqs. (12), (13) and (14) for the adsorption of MB along with the CR dyes over the CQDs and presented in Table 4 and Fig. 6. The Gibbs free energy (ΔG) negative values obtained suggests the spontaneity of the CR and MB adsorption over CQDs. The temperature-dependent value of ΔG indicates that the dyes' adsorption process on CQDs was more favourable at a slightly higher temperature^11^, which is consistent with the outcomes of the batch adsorption study for temperature optimisation. The ΔG values associated with the adsorption process that were evaluated from 298 to 323 K are directly related to the feasibility^13,15^. Since the data on the enthalpy and entropy changes are irrelevant in this research, the values for ΔH and ΔS for the 298–323 K temperature were excluded in this research^68–70^. The exothermic nature of the adsorption mechanism was revealed by the negative values of ΔH, and the reduced randomness at the solid/solution (adsorbent–adsorbate) interface during the sorption process was disclosed by ΔS negative’s value. In comparison to the initial state, the transition state is predicted to be more ordered by the negative values of ΔS°.

**Table 4:** Thermodynamics parameters for the CR and MB adsorption by CQDs.

Temperature (K)	ΔH (kJ mol^−1^)		ΔS (kJ mol^−1^)		ΔG (kJ mol^−1^)	
	MB	CR	MB	CR	MB	CR
298	− 1.695	− 1.431	− 0.122	− 0.165	− 0.389	− 1.102
303	–	–	–	–	− 0.394	− 1.135
313	–	–	–	–	− 0.395	− 1.140
323	–	–	–	–	− 0.399	− 1.141

### Mechanism of photocatalytic degradation using CQDs

**Figure 7:**
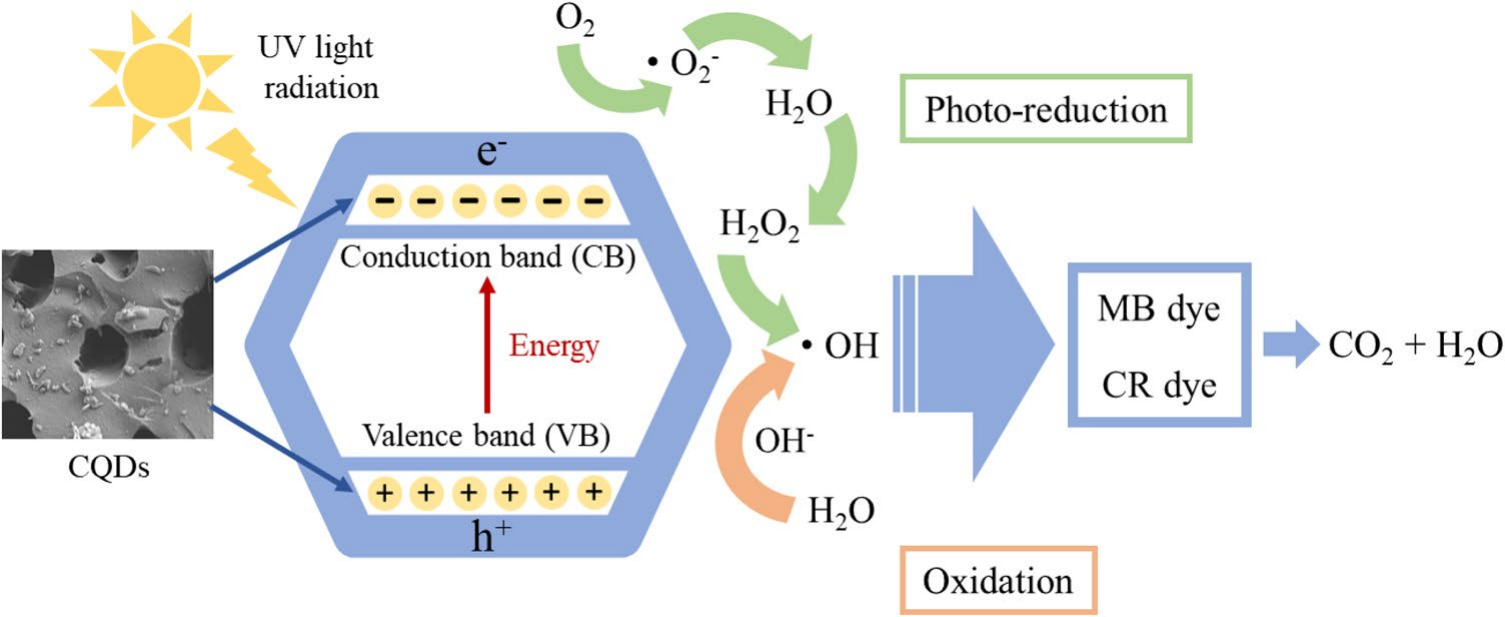
A possible mechanism for the photocatalytic degradation of the CR and MB dyes over CQDs photocatalyst.

In the current study, the synthesized CQDs were used as a photocatalyst in the photocatalytic degradation of dyes to aid and improve the process. Various functional groups on the surfaces of CQDs allowed electrostatic interactions to be the primary mechanism for degrading organic dyes, along with other contributing mechanisms for the adsorption^12,15,67^. The mechanistic interactions of MB and CR onto the CQDs are demonstrated in Fig. 7. The valence and conduction bands produce electron–hole pairs when exposed to sunlight irradiation, which results in the migration of holes (h^+^) and electrons (e^−^) to the CQDs’ surfaces for the reduction and oxidation processes^71,72^. The oxidation and photo-reduction processes produced the formation of free radical molecules, which is very reactive, attacking and degrading dye molecules^23^. In addition, whereas the valence band contains holes that will combine with water to form the hydroxyl radical, the conduction band has electrons that will react with oxygen to form superoxide. The produced reactive species continued to degrade the dyes. These results aligned with the optical analysis by UV–vis earlier.

### Photocatalytic degradation by CQDs

**Figure 8:**
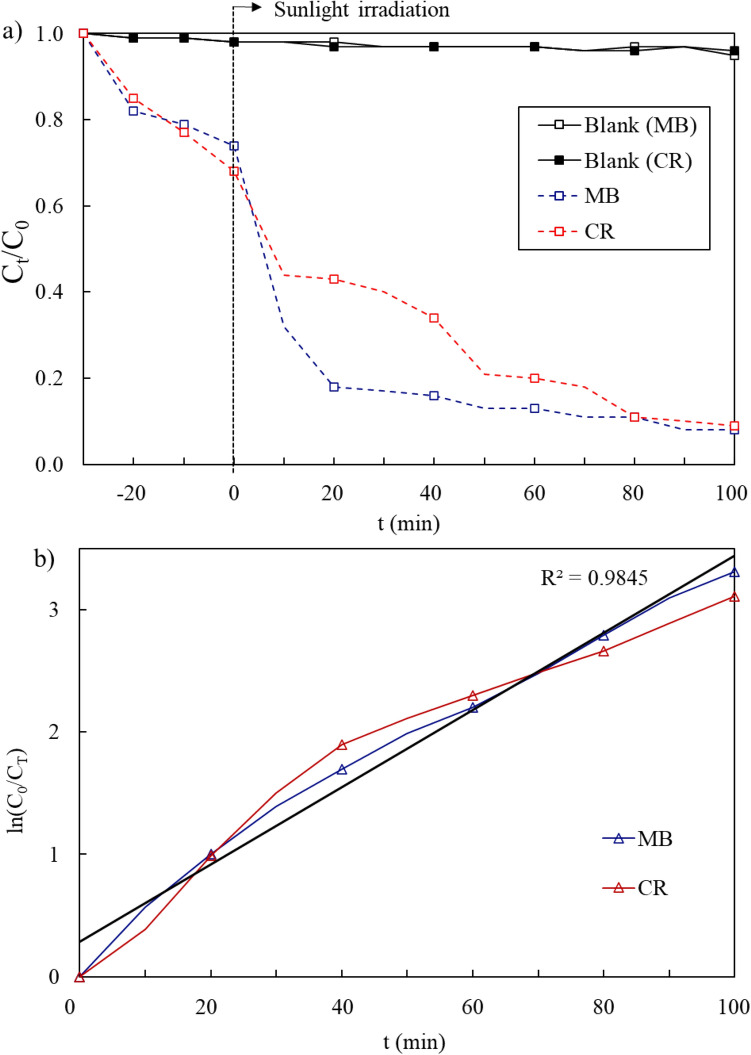
(**a**) Photocatalytic degradation of the dyes under sunlight irradiation; and (**b**) kinetic study of the dyes photodegradation.

The photocatalytic process of CQDs was investigated through the degradation of CR and MB under sunlight irradiation. Every sample was examined for adsorption equilibrium before the photocatalytic activity test. It can be understood from Fig. 8a that the CQDs showed 20% of MB adsorption after 30 min (dark reaction), while the CQDs showed about 30% CR removed by the adsorption. This is caused by the capacity of carbon-based quantum dot materials to have large surface areas, as well as the existence of carbonaceous species that are high in electronegative O atoms and sites of nitrogen acceptor in CR and MB molecules, which have a predilection for organic dye molecules^24,39,71^. After 20 min of treatment, the CQDs exhibited higher dye adsorption ability and displayed a further outstanding decolourization of MB and CR dyes. Although CQDs had limited capacity for dark reaction adsorption, the removal of dyes was nonetheless impressive because having a little amount of dyes adsorbed on the surface might act as a dye sensitizer, causing photosensitised degradation of dyes on the surfaces^39,73,74^. Amongst the organic dyes, because of their magnified photocatalytic activity as a result of their intense sunlight absorption, CQDs exhibited the best capacity to remove MB from the solution, along with the assistance from functional groups that make up the aromatic rings of MB, (C–S), (CN) and (CN=$-$), also known as a heterocyclic aromatic chemical compound. The sulphur atoms in the MB structure facilitated the degradation of the MB under sunlight irradiation, even without CQDs, because as the lone pairs in sulphur atoms were exposed to sunlight, they became active^23,41,75^. Due to the instability caused by repulsive forces, they reacted with the surface-adsorbed water molecules to form a stable bond in which a reaction between MB molecules and water took place^65^. After 90 min of reactions, CQDs have removed more than 90% of the MB.

After 10 min of treatment, the CQDs showed a further remarkable decolourization of CR dye and exhibited higher dye adsorption ability. Although CQDs had limited capacity for the dark reaction adsorption for CR, the removal of CR was nonetheless excellent though it was a bit lower than MB adsorption. As a result of its strong sunlight absorption, along with the two azo bonds (–N=N–) since CR is an azo dye, chromophore in its molecular structure, the fragmentation of the azo connections was interrupted during the photodegradation, which gave rise to the bleaching effect. Along with this efficient bleaching effect, this reaction was also thought to be proof of the destruction of aromatic fragments in the dye molecule and its intermediates^49,66^. It is demonstrable that the photocatalytic activity of CQDs under sunlight irradiation caused the CR dye to degrade. Nonetheless, the rate of degradation accelerated with the help of CQDs acting as photocatalysts in the presence of sunlight irradiation. This is because CQDs have distinct states that can yield unique fluorescent characteristics in which the degradation process is enhanced, and due to this fact, the lone pair electrons target the MB dye structure during the photocatalytic activity^23,76^.

The first-order kinetic model, where ln C = − kt + lnC_0_, in which k was the reaction rate constant, C_0_ and C were the concentration of CR or MB at initial and time t, was expressed by the Langmuir–Hinshelwood (L–H) model and used to ascertain the photocatalytic degradation reaction of MB and CR dyes. Figure 8b shows the linear plot; ln (C_0_/C_T_) versus the time for photodegradation of the dyes. Additionally, the results demonstrate that the photocatalytic degradation of MB and CR obeys PSO kinetics, as evidenced by the linear dependence (R_2_) of 0.9845 and a rate constant (K) of 0.06943 min^−1^. It is apparent from Fig. 8b that the first-order kinetic model fits the CQDs responses the best. The reaction rate constants, k was calculated to be 1.55 × 10^–2^ and 1.41 × 10^–2^ min^−1^ for both MB and CR dyes, respectively. The fastest photodegradation was seen in MB when compared to the CR dye.

Table 5 displays the results from other published studies using different adsorbents to remove MB and CR. A study by Chen et al.^11^ stated that MB was adsorbed 99.5% using carbon quantum dot-carbon nanotube composite (CQD-CNT) adsorbents, the highest among other studies reported. In this study, CQDs adsorbed 75% of the MB and 77% of the CR via batch adsorption, which is average compared to other studies. Hence, this study analysed the photocatalytic degradation performance of CQDs to the dyes. Maruthapandi et al.^25^ reported that almost all of the CR dye degraded (photocatalytic degradation) using polyaniline–nitrogen-doped carbon dot. CQDs derived from rubber seed shells synthesized in this study showed outstanding removal percentages and degradation rates, which align with previously published studies. As demonstrated in Table 5, the highest adsorption capacity in our investigation was equivalent to various earlier experiments. The outcomes suggested that the adsorption method for the dyes by CQDs was desirable. CR is typically easier to degrade or remove than MB dye, which in literature^25,77^ shown, some studies stated that dyes containing –N=N groups deteriorate more quickly than other forms of dye and are more vulnerable to being attacked by reactive species^78,79^. The kinetic rate or degradation/removal percentage of CR dye with two azo groups is, therefore, more significant than that of MB dye.

**Table 5:** Comparison of CQDs or quantum dots-based adsorbents in removing dyes reported in the literature.

Adsorbate	Adsorbent	Method	Removal (%)	References
MB	N, S doped carbon quantum dots	Adsorption	97	^10^
MB	Carbon quantum dot-carbon nanotube	Adsorption	99	^11^
MB	Nitrogen-doped carbon quantum dots	Photocatalytic degradation	97	^23^
MB	Polyaniline–nitrogen-doped carbon dot	Photocatalytic degradation	60	^25^
MB	Nitrogen-doped carbon quantum dots	Photocatalytic degradation	99	^46^
MB	ZnS quantum dots	Photocatalytic degradation	90	^65^
MB	Carbon dots	Photocatalytic degradation	85	^77^
MB	This study	Photocatalytic degradation	95	–
MB	This study	Batch adsorption	95	–
CR	Polyaniline–nitrogen-doped carbon dot	Photocatalytic degradation	99	^25^
CR	Chitosan/nano-CdS composite	Photocatalytic degradation	86	^26^
CR	Zinc sulphide quantum dots	Photocatalytic degradation	81	^66^
CR	Carbon dots	Photocatalytic degradation	90	^77^
CR	ZnO-modified SiO_2_ nanospheres	Adsorption	83	^80^
CR	This study	Photocatalytic degradation	93	–
CR	This study	Batch adsorption	97	–

### Stability of CQDs/regeneration

The stability and reusability of the adsorbents are crucial factors from an environmental and economic viewpoint. In this study, the reusability and stability of the CQDs were evaluated for the photocatalytic degradation of CR and MB dyes, along with the batch adsorption of the dyes. With a further increasing number of consecutive cycles, the degradation percentage of the dyes stayed above 85% until the eighth cycle. The CQDs demonstrated consistent performance, which proved that CQDs possessed an outstanding fluorescent property due to the changes in surface charges during the photodegradation reactions^11,23^. However, the degradation rate gradually decreased after the eighth cycle until the tenth, possibly due to the loss of photocatalytic active sites^13,15,81^. The same goes with the removal via adsorption; the percentage of CR and MB dyes remained above 75% until the eighth cycle and decreased slightly with a further increasing number of cycles. The change in adsorption capability may be due to the lower surface-active sites and strong binding between the adsorbents and the adsorbates themselves^44,75^. The high porosity of the synthesized adsorbents may be responsible for the adsorbents' capacity for recyclability and the excellent regeneration properties of the CQD adsorbents. After repetitive exposure to UV light, the CQDs' photoluminescence remained the same, indicating CQDs' good stability. From this point of view, these results can confirm the relative stability and reusability of the CQDs and could easily be regenerated, in which could be maintained for at least eight cycles.

## Conclusions

Carbon quantum dots derived from biomass were prepared as an effective adsorbent and a catalyst for photocatalytic degradation of CR and MB dyes under sunlight irradiation, along with adsorption of the dyes, under the operational condition of dosage (0.02–0.1 g), temperature (25–55 °C), contact time (2–20 min), and pH (2–12). The CQDs were subjected to characterization XRD analysis, Raman spectroscopy, FTIR spectroscopy, P–L spectroscopic, UV–visible spectroscopy, SEM, EDX mapping and TEM. These analyses confirmed the relatively rough and uneven porous surface morphology while still being slightly uniform and the closely aligned distribution of pores. The CQDs were observed to be a multi-layered adsorbent and exhibited intrinsic properties as a high-quality CQDs adsorbent though they still possess defects in the material. Moreover, the peaks showed in Raman spectra validated the presence of the lattice distortions, and FTIR analysis proved the existence of carboxyl, hydroxyl and carbonyl groups attached to the CQDs structures’ aromatic ring, which indicated the significance of the oxygenated functional groups and discovered the efficacious oxidation of raw materials. Photoluminescence test revealed high excitation intensity of the CQDs, subsequently leading to higher energy emissions by the material, therefore possessing a luminescent property. Under UV radiation, the CQDs possess greenish-blue fluorescence that aids the photocatalytic redox reaction.

Langmuir isotherm and PSO models effectively captured the nature of CR and MB adsorption onto the CQDs, indicating that monolayer adsorption mechanisms were favoured. There was only a slight difference in the percentage of dyes removed between photocatalytic degradation and batch adsorption methods, with 93% and 77%, respectively, for CR and, 94% and 75% for MB. Thermodynamics study suggested that the adsorption process was spontaneous and exothermic. Besides, CQDs also proven excellent reusability and stability. The photocatalytic degradation process involved multiple mechanisms, including hydrogen bonding, electrostatic interactions, π–π interactions, pore-filling, ion exchange and photocatalytic redox reaction. Under sunlight irradiation, CQDs' photocatalytic degradation efficiency on both dyes was greater than 90% and obeyed the first-order kinetic L–H model. This study found that CQDs derived from biomass can be considered excellent adsorbents and outstanding photocatalysts for degrading organic dyes. However, to address the existing problems with water quality, it is necessary to investigate and analyse the efficacy of various local biomass wastes as well as the possibility for scaling up CQDs into larger production. In order to create wastewater treatment technologies that are environmentally responsible, it is also essential to further investigate the total energy needed from scale-up lab research to a full-scale commercial production. In addition, CQDs derived from biomass can be furthered decorated with other materials to form multifunctional composites while being robust for future research.

## Materials and methods

### Materials and chemicals

In this research, rubber seed shells were obtained from a local plantation in Malaysia. Sulphuric acid (H_2_SO_4_) (95–97%), potassium permanganate (KMnO_4_) (> 99%), sodium hydroxide (NaOH) (98%), citric acid monohydrate (CA ≥ 99.5%), hydrochloric acid (HCl) (99%), CR, MB with ≥ 97.0% (analytical grade) and ammonia solution (25%) were attained from Sigma-Aldrich, Malaysia. Except as otherwise stated, all substances employed in the research were of the analytical grade. These substances were not further processed or purified before usage. 18.2 MΩ ultrapure deionized water from pureLab Flex was used to prepare all aqueous solutions.

### Fabrication of carbon quantum dots (CQDs)

CQDs were fabricated from graphene oxide (GO) derived from rubber seed shells (biomass waste) using the previously utilized used a slightly modified version of Hummers' approach^13,15^, where the graphite surface was functionalized by introducing functional groups in the presence of the potassium permanganate (KMnO_4_) reagent, which was oxidized by the addition of H_2_SO_4_ and KMnO_4_ in a 1:4 ratio. Prior to the extraction of CQDs, the rubber seed shells were smashed into smaller sizes, after which they were thoroughly washed using deionized water to remove impurities on the surface. The precursor was then dried for 8 h at 200 °C in a muffle furnace. The dried precursor was utilized for the surface functionalization of CQDs and for forming a carbon core, which was expected to possess high-intensity CQDs with effective luminescent properties^23,51^.

**Figure 9:**
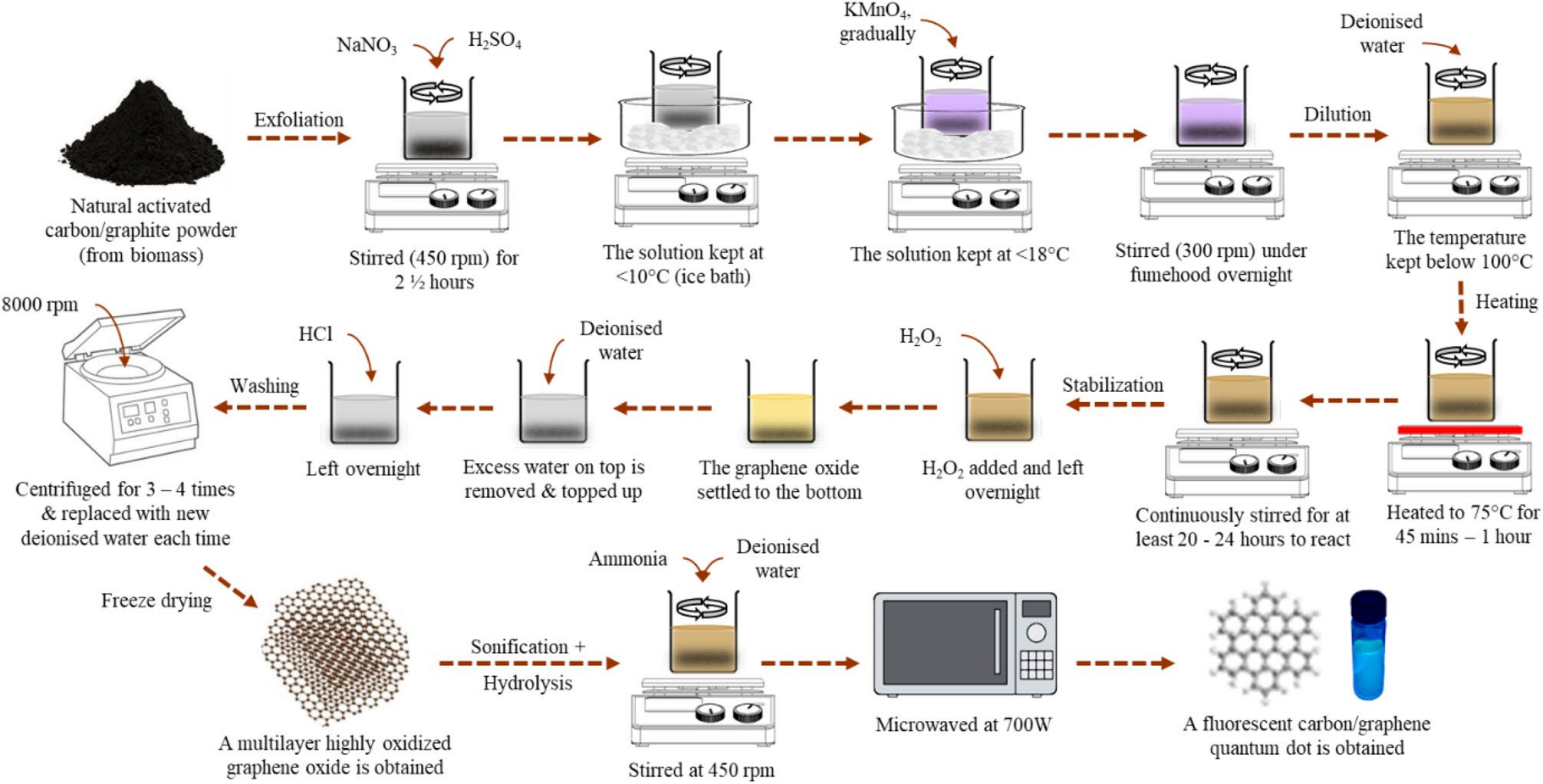
The synthesis process of carbon quantum dots (CQDs).

The process flow of the CQDs synthesis route is shown in Fig. 9. 30 mL of ultrapure water were initially used to dissolve the precursor. After stirring the mixture for 15 min, 20 mL of ammonia solution was added. After then, the solution was homogenized using a bath sonicator for 30 min. This was followed by heating the solution in the microwave, which was radiated for 30–120 s using a Sharp R239EK microwave at a medium power rating of 400 W. After the radiation was achieved, the solution was naturally cooled, after which the obtained deep yellowish-brown product was centrifuged for 15 min at a speed of 10,000 rpm to produce CQDs. Lastly, the obtained CQDs were stored in a container until it was utilized for further analysis. The photocatalysts were present in the concentrated solution that was produced following the fabrication procedure. To encourage UV light penetration before it reaches the surfaces of the catalyst, where photodegradation was anticipated to happen, the concentrated solution was diluted. However, the exact concentration of CQDs was not analysed in this work due to difficulties in quantifying the quantum dots' conversion rate from biomass.

### Characterization

The functional properties of the fabricated CQDs were analysed by FTIR Spectroscopy (Nicolet iS10). Gemini model 18 SUPRA 55VP-ZEISS Oberkochen (Germany) FESEM model with energy-dispersive X-ray 19 spectroscopy was used to get the morphological surface mapping of CQD (EDX). Scanning electron microscopy was used to examine the surface morphology (Hitachi S-3400N), and transmission electron microscopy (Thermo Fisher; Talos 120C) was used to acquire the nanostructure, including the CQDs' particle size. The optical properties of the material and the degradation potentials of the pollutants were determined using UV–vis spectrophotometry (UV260, Shimadzu) over a range of 200–800 nm. A nano-zeta potential analyser (Malvern) was used to examine the hydrodynamic characteristics of CQDs. The emission was examined using photoluminescence spectroscopy (FLS920 Edinburgh Instrument), and a detection range of 200–1000 nm was used to acquire the excitation light. Lastly, the elemental composition of the CQDs was obtained using the energy-dispersive X-ray analysis (Apollo X). The adsorbent's structure, composition and material properties were obtained using XRD DIFFRAC EVA software and Bruker AXS D8 Advance Karlsruhe Germany. Unless otherwise stated, all measurements were made in ambient circumstances.

### Adsorption-photocatalytic degradation of organic dyes

The synthesised CQDs were used to adsorb organic dyes while maintaining a constant temperature in a double-walled Pyrex glass chamber and the solution was stirred using a magnetic stirrer. In order to achieve equilibrium between adsorption and desorption, the adsorption-photocatalytic processes were conducted in the dark^82^. The adsorptive ability of the synthesized CQDs was examined using a batch adsorption method. During a 20-min contact period, 20 mg of CQDs were added to several flasks holding 30 ml of CR and MB solutions (starting concentration: 20–120 mg L^−1^). 0.1 N HCl and KOH were used to adjust the supernatant's pH. At temperature intervals between 298 and 323 K, the effects of concentration, adsorbent dose, solution pH, and contact duration were investigated. According to Eq. (1), the removal percentages of MB and CR (q%) were assessed. Equation (2) was used to assess the adsorption capabilities of CQD for the adsorption of the studied contaminants. After being desorbed, the dye-loaded adsorbents underwent regeneration (0.1 M HCl). The samples were oven dried at 80 °C for 8 h after being washed with deionized water to get rid of any remaining dye residues. In 30 mL of 100 mg L^−1^ pollutant solutions, 50 mg of CQDs were dye-loaded and incubated for 30 min at 298 K. The regenerated adsorbents' ability to adsorb and remove MB and CR was calculated. Using Eq. (3), the removal percentages were computed. The adsorbed CQDs were then subjected to regeneration until all contaminants had been fully desorbed:

1 $$\mathrm{Removal percentage}:q\%=\frac{({{C}_{0}-C}_{e})}{{C}_{0}}\times 100\%$$

2 $$\mathrm{Uptake capacity}:{q}_{e}=\frac{({{C}_{0}-C}_{e})V}{M}$$

In which q% stands for the total removal percentage, C_0_ (mg L^−1^) for pollutant concentration before to adsorption, Ce (mg L^−1^) for pollutant concentration following adsorption, qe (mg g^−1^) for adsorption capacity, V (L) for solution volume, and M (g) for adsorbent mass. The ability of CQDs as photocatalysts for the degradation of organic dyes was assessed by evaluating their degradation efficiency using MB and CR solutions under the influence of sunlight irradiation. Before this was done, to make sure that the reactants and CQDs attained adsorption and desorption equilibrium, the solution sample was left in the dark for 120 min^23^. The solution was left in the sun for up to 160 min, and as a control, dye solutions without CQDs were also left outside in the sun. A UV–vis spectrophotometer was used to quantify and evaluate the deteriorated dye solutions. Using Eq. (3), it was possible to compute the percentage of dye degradation^21^:

3 $$ {\text{Degradation }}\left( \% \right):\left[ {\left( {{\text{A}}_{0} {-}{\text{A}}_{{\text{t}}} } \right)/{\text{A}}_{0} } \right] \times {1}00\% $$

where A_t_ is the dye's absorbance at a certain time and A_0_ is the dye's initial absorbance. A UV–vis spectrophotometer was used to measure the absorbance values of MB and CR at 660 nm and 496 nm, respectively. This study also included a photocatalytic kinetic analysis to assess the kinetics of the photodegradation rate, which was generated using the first-order kinetics model (Eq. 4)^83^:

4 $$ {\text{First - order kinetics model}}:\ln \left( {A_{0} /A_{t} } \right) = kt $$

where A_0_ is the initial dye absorbance, A_t_ is the dye absorbance at the time, and k is the reaction rate constant calculated from the graph's slope (t). CQDs were utilized as a liquid photocatalyst in this investigation because of their ability to disperse easily in an aqueous solution. Upon the degradation study, CQDs could be isolated from the dyes that had degraded, allowing them to be reused in the next cycles. By subjecting the CQDs solution to UV light irradiation, the fluorescence of the solution was examined (302 nm) and to further examine the effectiveness of CQDs in photodegradation activity, the photocatalytic degradation of MB and CR dyes was carried out using CQDs as photocatalysts.

### Adsorption isotherms

Adsorption isotherm models were employed to use equilibrium isotherm equations to describe the adsorption process of the dyes^12,13,15,44^. In this study, the Langmuir, Freundlich, and Dubinin–Radushkevich isotherm models were used to fit the isothermal experiment data according to Eqs. (5–7):

5 $$\mathrm{Langmuir isotherm}:\frac{1}{{q}_{e}}=\frac{1}{{q}_{m}}+\frac{1}{{K}_{L}{q}_{m}{C}_{e}}$$

6 $$\mathrm{Freundlich isotherm}:log{q}_{e}=log{K}_{f}+\frac{1}{n}log{C}_{e}$$

7 $$\mathrm{Dubinin}-\mathrm{Radushkevich isotherm}:ln{q}_{e}=ln{q}_{m}+ \beta {\varepsilon }^{2}$$

where the theoretical maximum sorption value is represented by qm (mg g^−1^), the Langmuir coefficient is represented by K_L_ (L mg^−1^), the Freundlich coefficient is represented by n, and the Dubinin–Radushkevich coefficient is represented by β. Initial concentrations for MB and CR under the same operating conditions were 20, 40, 60, 80, 100, and 120 mg L^−1^ in isothermal adsorption tests. For analysis, the aqueous samples were obtained at equilibrium.

### Adsorption kinetics

The latent rate-limiting steps and the adsorption rate^12,13,15,44^ were studied using adsorption kinetics where the pseudo-first-order (PFO), pseudo-second-order (PSO), intraparticle diffusion, and Elovich models were fitted to the kinetics data, as listed according to Eqs. (8–11):

8 $$\mathrm{PFO model}:\mathit{ln}({q}_{e}- {q}_{t}) = \mathit{ln}{q}_{e}- {k}_{1}t$$

9 $$\mathrm{PSO model}:\frac{t}{{q}_{1}}=\frac{1}{{K}_{2}{q}_{e}^{2}}+\frac{1}{{q}_{e}}(t)$$

10 $$\mathrm{Intraparticle diffusion model}:{q}_{t}={k}_{3}{t}^{ 0.5}+ C$$

11 $$\mathrm{Elovich model}:{q}_{t}={k}_{i}{t}^{0.5}+ c$$

where C (mg g^−1^) is the constant of the intraparticle diffusion model, q_e_ is the equilibrium concentration of adsorbate in a solution, q_t_ is the adsorbed adsorbate at time t, k_1_ (1/min), K_2_ (g/(mg min)), and k_3_ (mg/(g min^1/2^)) are the rate constants of adsorption. The PSO kinetic (Elovich equation), where k_i_ stands for the intraparticle diffusion constant and C being the intercept, assumed that the real media surfaces were heterogeneous depending on their energy.

### Thermodynamics study

The Gibbs free energy change (ΔG), enthalpy change (ΔH), and entropy change (ΔS), which are thermodynamic parameters, were employed to describe the adsorption process’ nature for dyes on the surface of CQDs, in which Eqs. (12) and (13) were used to determine the parameters' values at various temperatures. T (K) is the absolute temperature, R (8.314 J mol^−1^ K^−1^) is the universal gas constant and K_d_ is the dissociation constant. The y-intercept and slope of Eq. (14) were used to compute the ΔS, ΔH, and ΔG values for the MB and CR solutions:

12 $$\Delta G= - RT ln {K}_{d}$$

13 $$\Delta G=\Delta H-T\Delta S$$

14 $$\mathit{ln}K= \frac{\Delta S}{R}-\frac{\Delta H}{RT}$$

### Reduction kinetics of the dyes

A UV–visible spectrum was used to evaluate the photocatalytic activity of the catalyst (CQDs) on the degradation of the dyes when it was exposed to direct sunlight at various intervals. The reduction kinetics of the dyes have been investigated using the Langmuir–Hinshelwood (L–H) dynamic model.

15 $$dc/dt=kKc/(1+Kc)$$

16 $$\mathit{ln}(\frac{{C}_{0}}{{C}_{T}})=kt$$

where c represents the concentration at any given time, k and K represent the equilibrium constant and the reaction's limiting rates at maximum coverage under the given experimental conditions. When the substrate concentration is less than one, Eq. (15) may be changed into a PFO kinetic equation, as shown in Eq. (16). C_T_ and C_0_ stand for dye concentrations at time, t and equilibrium adsorption respectively, while k is the constant of apparent rate^38^. Therefore, the concentration of C_0_ has no effect on the rate of reaction. The rate of photocatalytic degradation of the dyes can be fitted linearly to the slope of the ln (C_0_/C_T_) against time. The PFO kinetics and the L–H dynamic model are both employed to explain how the dyes photodegraded on these photocatalysts. This is demonstrated by the linear relationship between ln (C_0_/C_T_) and t.

### Ethical approval

There are no experimental research and field studies on plants (either cultivated or wild), including the collection of plant material in this study. If there is, it must comply with relevant institutional, national, and international guidelines and legislation.

## Data Availability

The datasets used and/or analysed during the current study available from the corresponding author on reasonable request.
